# Development and Degeneration of Cone Bipolar Cells Are Independent of Cone Photoreceptors in a Mouse Model of Retinitis Pigmentosa

**DOI:** 10.1371/journal.pone.0044036

**Published:** 2012-08-31

**Authors:** Miao Chen, Ke Wang, Bin Lin

**Affiliations:** 1 Department of Anatomy, The University of Hong Kong, Li Ka Shing Faculty of Medicine, Pokfulam, Hong Kong, People's Republic of China; 2 Eye Institute, The University of Hong Kong, Li Ka Shing Faculty of Medicine, Pokfulam, Hong Kong, People's Republic of China; 3 State Key Laboratory of Brain and Cognitive Sciences, The University of Hong Kong, Li Ka Shing Faculty of Medicine, Pokfulam, Hong Kong, People's Republic of China; University Zürich, Switzerland

## Abstract

Retinal photoreceptors die during retinal synaptogenesis in a portion of retinal degeneration. Whether cone bipolar cells establish regular retinal mosaics and mature morphologies, and resist degeneration are not completely understood. To explore these issues, we backcrossed a transgenic mouse expressing enhanced green fluorescent protein (EGFP) in one subset of cone bipolar cells (type 7) into *rd1* mice, a classic mouse model of retinal degeneration, to examine the development and survival of cone bipolar cells in a background of retinal degeneration. Our data revealed that both the development and degeneration of cone bipolar cells are independent of the normal activity of cone photoreceptors. We found that type 7 cone bipolar cells achieved a uniform tiling of the retinal surface and developed normal dendritic and axonal arbors without the influence of cone photoreceptor innervation. On the other hand, degeneration of type 7 cone bipolar cells, contrary to our belief of central-to-peripheral progression, was spatially uniform across the retina independent of the spatiotemporal pattern of cone degeneration. The results have important implications for the design of more effective therapies to restore vision in retinal degeneration.

## Introduction

Cone bipolar cells, a large population of the second order neurons in the mammalian retina, are the essential backbone of cone pathways, which relay visual information from photoreceptors in the outer retina and synapse onto the third-order retinal neurons, ganglion and amacrine cells in the inner retina [1], [2]. There are nine morphological subsets of cone bipolar and one type of rod bipolar cells in the mammalian retina [3]. Functionally, bipolar cells are subdivided into two major functional classes: ON cells and OFF cells. ON bipolar cells that respond to light increments have axons terminating in the inner half of the inner plexiform layer (IPL), whereas OFF bipolar cells that respond to light decrements have axons which stratify in the outer half of the IPL [4], [5], [6], [7]. In retinitis pigmentosa (RP), photoreceptors die. RP is a family of diseases in which a mutation results in death of rod photoreceptors and subsequent cone death at a slower rate [8], [9], [10], leading to functional blindness with bipolar cells left without their major source of input. The development of new treatments for curing retinal degeneration, such as gene therapy and cell transplantation, is heavily based on the assumption that bipolar cells and their underlying synaptic circuits in the inner retina remain relatively unaffected during and after photoreceptor death in RP. However, there is increasing evidence that the secondary degeneration in remaining neurons occurs, such as in rod bipolar cells and horizontal cells [11], [12], [13], [14], [15]. Due to a lack of antibodies that might specifically label cone bipolar cells, it is not completely understood about the impact of cone loss on the development of spatial organization, the maturation of axons and dendrites, and the maintenance of cone bipolar cells during retinal degeneration. It is of great interest to know the status of cone bipolar cells in RP and to identify the practical windows of opportunity for more effective therapies that would help to prevent vision loss.

Here we applied a method that did not use immunocytochemistry to study the development and degeneration of cone bipolar cells in a classic *rd1* mouse model of RP, in which degeneration overlaped with retinal synaptogenesis [8], [9]. We backcrossed transgenic GUS8.4-GFP mice [16], [17], in which populations of one subset of cone bipolar cells expressed green fluorescent protein (GFP), into *rd1* mice to create mice that expressed both a mutant *rd1*gene and a reporter gene. The entirety of cone bipolar cells in the resulting mouse line was visualized with a degree of detail through the expression of EGFP that cannot readily be achieved by immunolabeling methods. This method made it possible for us to directly observe early development and late responses to pathological stimuli on the single-cell level in cone bipolar cells.

## Materials and Methods

### Animals

Two lines of mice, transgenic GUS8.4GFP mice (357 mice) carrying the gene for GFP driven by an 8.4-kb promoter region upstream from the gustducin gene [16], [17] and C3H/HeJ mice, homozygous for *rd1* mutation (*rd1/rd1*) (The Jackson Laboratory), were used for experiments. In addition, we generated a line of 357 / *rd1* mice by crossing 357 mice with *rd1* mice. The 357 mouse lines were mated with *rd1* mice for more than six generations. Genotyping was performed by PCR on tail-extracted DNA to identify 357-GFP positive animals. The following primers were used: 357-GFP F (AAGTTCATCTGCACCACCG) and 357-GFP R (TCCTTGAAGAAGATGGTGCG). To identify mice homozygous for the *rd1* mutation among 357-GFP positive individuals, a second PCR was performed. In this case, the primers were: *Rd1* -F (CTTTCTATTCTCTGTCAGCAAAGC) and *rd1*-R CATGAGTAGGGTAAACATGGTCTG), following a protocol recommended by the Jackson Laboratories. Experimental procedures were in accordance with institutional guidelines and with the ARVO statement for the use of animals in research. All mice were kept in a local facility in a 12-hour light/dark cycle with illumination levels below 60 lux.

### Immunocytochemistry and Imaging

Animals were anesthetized with the mixture of ketamine hydrochloride (30–40 mg/kg) and xylazine (3–6 mg/kg). Eyes were quickly enucleated after a reference point was taken to label superior poles and retinas were dissected free of the vitreous and sclera in carboxygenated Ames' Medium (Sigma, St. Louis, MO). Retinas were fixed in 4% paraformaldehyde (PFA) for 0.5–1 hour and then blocked for 1 hour in a solution containing 4% normal goat serum (NGS), 1% bovine serum albumin (BSA), and 0.5% Triton X-100 in phosphate-buffered saline (PBS; pH 7.4). Some of retinas were sectioned serially at 10–12 µm along dorsoventral axe on a vibratome. The following primary antibodies were applied: rabbit anti-GFP (1∶500, Molecular Probes, Eugene, OR); mouse anti- protein kinase C (PKCα) clone MC5 (1∶200, Amersham, Arlington Heights, IL); mouse anti- C-terminal binding protein 2 (CtBP2) (1∶200, BD Biosciences, San Jose, CA); rabbit anti-red/green opsin (1∶500, Chemicon Temecula, CA) and rabbit anit- calcium-binding protein 5 (Cabp5) (1∶500, kindly provided by Dr. Haeseleer, Department of Ophthalmology, Seattle, WA). The primary antibodies were diluted in 2% NGS, 1% BSA, 0.5% Triton X-100 in PBS and applied overnight. After several washes in PBS, secondary antibodies conjugated either to Alexa 488 or 594 (1∶500; Molecular Probes, Eugene, OR) were applied for 2 hours. Tissues were mounted in Vectashield (Vector Laboratories, Burlingame, CA).

Confocal micrographs of fluorescent specimens from both retinal flat-mounted preparations and cross sections were captured at a resolution of 1024–1024 pixels using a Zeiss LSM 700 Meta Axioplan 2 laser scanning confocal microscope (Carl Zeiss) equipped with argon and helium-neon lasers. Plan-Apochromat 63×/1.4 or 40×/1.4 oil immersion objective lenses were used. Through-focus image stacks were taken of every cell and processed using Metamorph software (Universal Imaging). Images scale was calibrated, and if necessary, brightness and contrast were adjusted using Photoshop CS3 software (Adobe Systems, San Jose, CA, USA).

### Data Analysis

The quantification of survival of type 7 cone bipolar cells at different ages was carried out in flat mounted retinas of 357/*rd1* mice. We counted all surviving type 7 cone bipolar cells, identified by the expression of GFP, in four 240 µm by 240 µm regions in the dorsal retina along the dorsal-ventral axis. To quantify both dendritic and axonal arbor sizes of type 7 bipolar cells, a convex polygon was drawn by connecting the distalmost tips of dendrites and axon terminals using the MetaMorph software (Universal Imaging) and the area was calculated. To facilitate comparison it was sometimes converted to equivalent diameter, by assuming a circular dendritic field.

The spatial organization of type 7 cone bipolar cells was investigated by analyzing the density recovery profile (DRP) as described previously [18]. The area sampled included roughly half of the test retina. Digital images of each field were collected and digitally montaged. Distances between cells were measured by a locally written computer program [19].

The quantification of survival of other subsets of cone bipolar cells at different ages was carried out in cross sections of 357/*rd1* mice stained with GFP and Cabp5, which labels populations of three subsets of bipolar cells (two subsets of cone bipolar cells and rod bipolar cells. Cabp5 positive bipolar cells were counted in three 240 µm by 240 µm regions in the dorsal retina of cross sections along the dorsal-ventral axis. Since Cabp5 antibody also labels rod bipolar cells, so we counted numbers of rod bipolar cells labeled by PKCα in the same regions and then deducted those cells that were positive for both PKCα and Cabp5 from Cabp5 positive cells.

### Statistics

Data were represented as means±SD. ANOVAs with Bonferroni’s and Dunnett’s *post hoc* tests for multiple comparisons were performed with Origin (OriginLab) and programs written in MATLAB (Mathworks) on full datasets to detect significant differences in the mean. P value <0.05 was considered statistically significant.

## Results

The major goal in the present study was to address questions of whether cone bipolar cells could achieve regular mosaics and develop a mature dendritic and axonal morphology in the absence of normal synaptic inputs from cone photoreceptors, as well as the neuronal depletion pattern of cone bipolar cells soon after in the *rd1* mouse model of RP. To explore these issues, we generated a line of mice by crossing a transgenic mouse, in which one subset of ON cone bipolar cells (type 7 ON cone bipolar cells) expressed a high level of GFP [16], [17], into *rd1* mice, and carried out investigation.

### Type 7 Cone Bipolar Cells Express GFP in the 357/rd1 Mouse Retina

**Figure 1:**
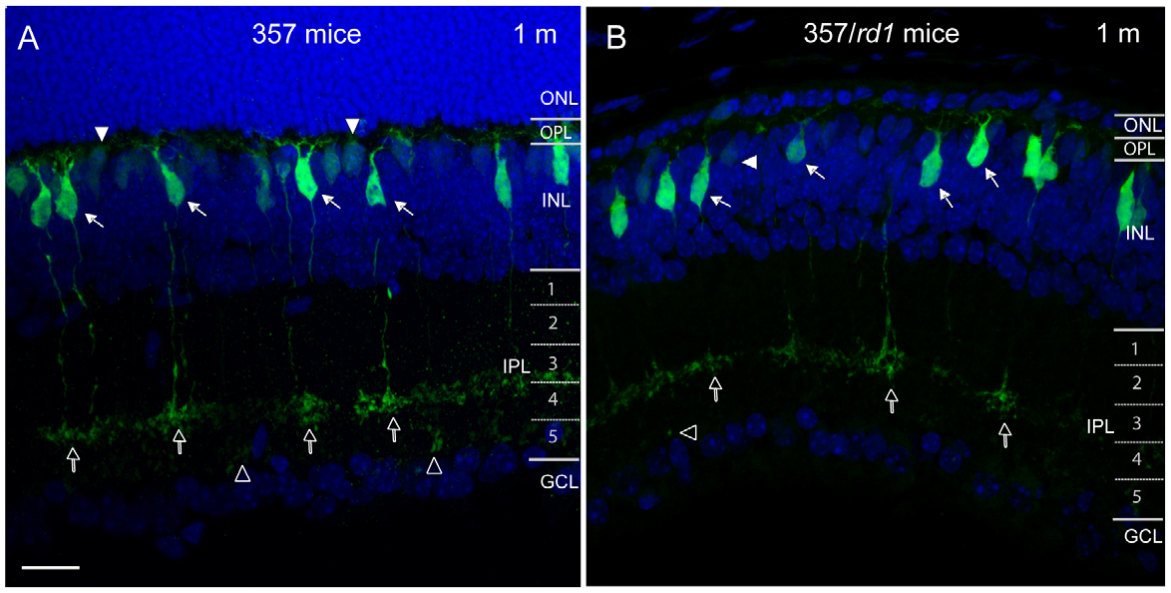
Cross sections of fixed tissue show type 7 cone bipolar cells express GFP in the retinas of age-matched transgenic 357 mice and 357/*rd1* mice. Type 7 bipolar cells (**A** and **B**, green, solid arrows) are seen in low power images, whose axonal arbors terminated in the OPL and dendritic arbor (open arrows) stratified in the stratum 4 of the IPL. Solid arrowheads point to faint labeling of rod bipolar cells, whose axonal arbors (**A** and **B**, open arrowheads) stratified in the stratum 5 of the IPL. To label the nuclear layers, we stained cell nuclei with diamidinophenylindole (DAPI) (**A**, blue). The ONL in the retina of 357/*rd1* mice was reduced to a single row by P30 (**B**, Blue). ONL, outer nuclear layer; OPL, outer plexiform layer; INL, inner nuclear layer; IPL, inner plexiform layer; GCL, ganglion cell layer. Scale bar, 20 µm.

Type 7 cone bipolar cells have been previously identified to express a high level of GFP in the GUS8.4GFP transgenic mouse line (357 mice) [16], [19]. The dendrites of type 7 bipolar cells stratifying in the outer plexiform layer (OPL), and axon terminals terminating in sublamina 4 of the inner plexiform layer (IPL), were prominently labeled in 357 mice (Figure 1A, solid arrows). Faint labeling of rod bipolar cells was also observed in the same line (Figure 1A, solid arrowheads). Type 7 cone bipolar cells can be easily distinguished from rod bipolar cells based on either the level of GFP expression, or the level of axonal stratifications. The axon terminals of type 7 bipolar cells terminated in sublamina 4 of the IPL (Figure 1A, open arrows), while the axon terminals of rod bipolar cells were located in sublamina 5 of the IPL, close to the ganglion cell layer (GCL) (Figure 1A, open arrowheads). To label nuclear layers, we stained cell nuclei with diamidinophenylindole (DAPI) (Figure 1, blue). The outer nuclear layer (ONL), where cell bodies of photoreceptors are located, was reduced to a single row in 357 / *rd1* mice by 1 month of age (Figure 1B, blue), and type 7 bipolar cells expressed GFP (Figure 1B, arrows). The visualization of fine-detailed morphology of type 7 bipolar cells was achieved by the high level of GFP expression. Thus, the 357 / *rd1* mouse line was a good model system for investigating early development and late degeneration on the single-cell level in type 7 ON cone bipolar cells.

### Type 7 Bipolar Cells Develop Regular Mosaics and a Normal Morphology in rd1 Mice

**Figure 2:**
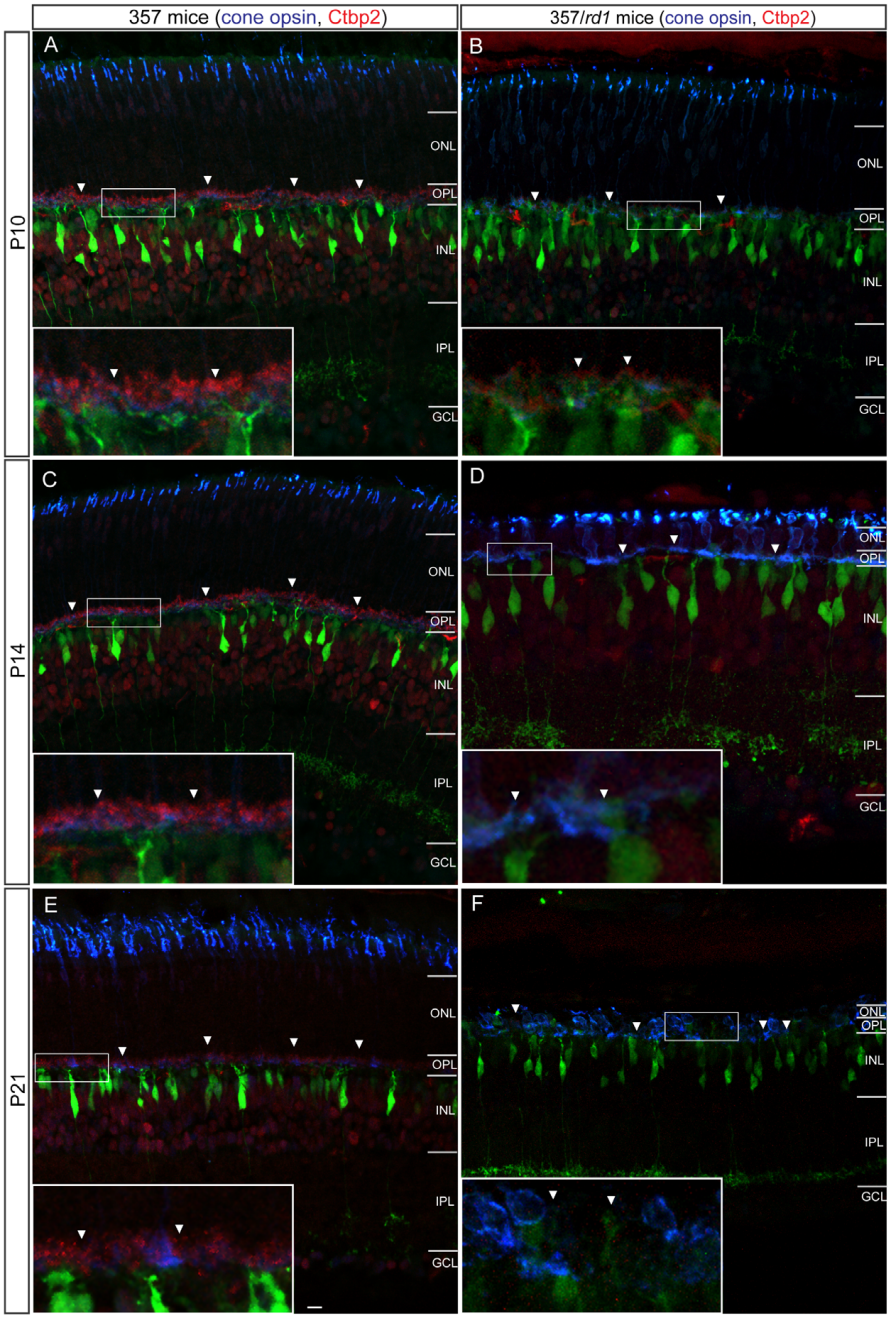
The correlation of the time course of cone degeneration and type 7 cone bipolar development in *rd1* mice. Counterstaining of retinal sections with antibodies against red/green opsins and Ctbp2 shows cone photoreceptors (blue) and synaptic ribbons (red) in the both OPL and IPL. Type 7 cone bipolar cell dendrites invaded cone terminals to form ribbon synapse complex starting from P8. By P10, a high density of synaptic ribbons were present in the OPL of WT mice (**A,** red, arrowheads), while few were observed in the OPL of *rd1* mice (**B,** red). Insets illustrate highly magnified image from the boxed regions. Cone photoreceptors did not appear to have a normal morphology by this age (**B,** blue) compared with their counterparts in WT (**A,** blue). At P14 (**C**, **D**), some cone photoreceptors lost OS and IS, axons and axonal terminals became much smaller in *rd1* retinas (**D,** blue) compared to their counterparts in WT (**C,** blue). Synaptic ribbons were barely observed in the OPL (**D,** red, arrowheads). By P21 (**E**, **F**), most cones lost OS, IS and axonal terminals, and the absolute number of S cones was decreased (**F,** blue). Synaptic ribbons were absent from the OPL by this age (**F,** red, arrewheads). Type 7 cone bipolar cells appeared identical in morphology between WT mice and *rd1* mice at each of age-matched retinas (green). ONL, outer nuclear layer; OPL, outer plexiform layer; INL, inner nuclear layer; IPL, inner plexiform layer; GCL, ganglion cell layer; ONH, optic nerve head; OS, outer segment; IS, inner segment. Scale bar, 20 µm.

Rod bipolar cells fail to develop normal dendritic arborization and to maintain their characteristic morphology at maturity in *rd1* mice [14], [20]. We set out to evaluate the correlation of the time course of cone degeneration and the maturation of dendritic and axonal arbors of type 7 bipolar cells, and also the development of regular mosaics in the same line of *rd* mice. Cone bipolar cells develop a mature morphology within first three weeks of postnatal life in the mouse retina [21], [22]. We thus collected retinas at three time points (P10, P14 and P21) right before its maturity at postnatal day 21 (P21) and labeled them with an antibody against cone opsins. Figure 2 shows temporal pathological changes of cone photoreceptors and the development of type 7 cone bipolar cells. Cone photoreceptors starts degeneration shortly after P8 [23], [24], the time when cone bipolar cells are under differentiation [21]. Consistent with our previous observation [24], we found that cones no longer had a normal morphology in the *rd1* retina by the age of P10 (Figure 2B, blue): some cone photoreceptors lost outer segment (OS), or had flattened inner segment (IS), compared to their counterparts in age-matched WT (Figure 2A, blue). With increasing age, the atrophy of processes in cone photoreceptor cells was more extensive (Figure 2D, blue). By P21, most cones lost OS, IS and axonal terminals, and the absolute number of cone photoreceptors was dramatically decreased as well (Figure 2F, blue).

**Figure 3:**
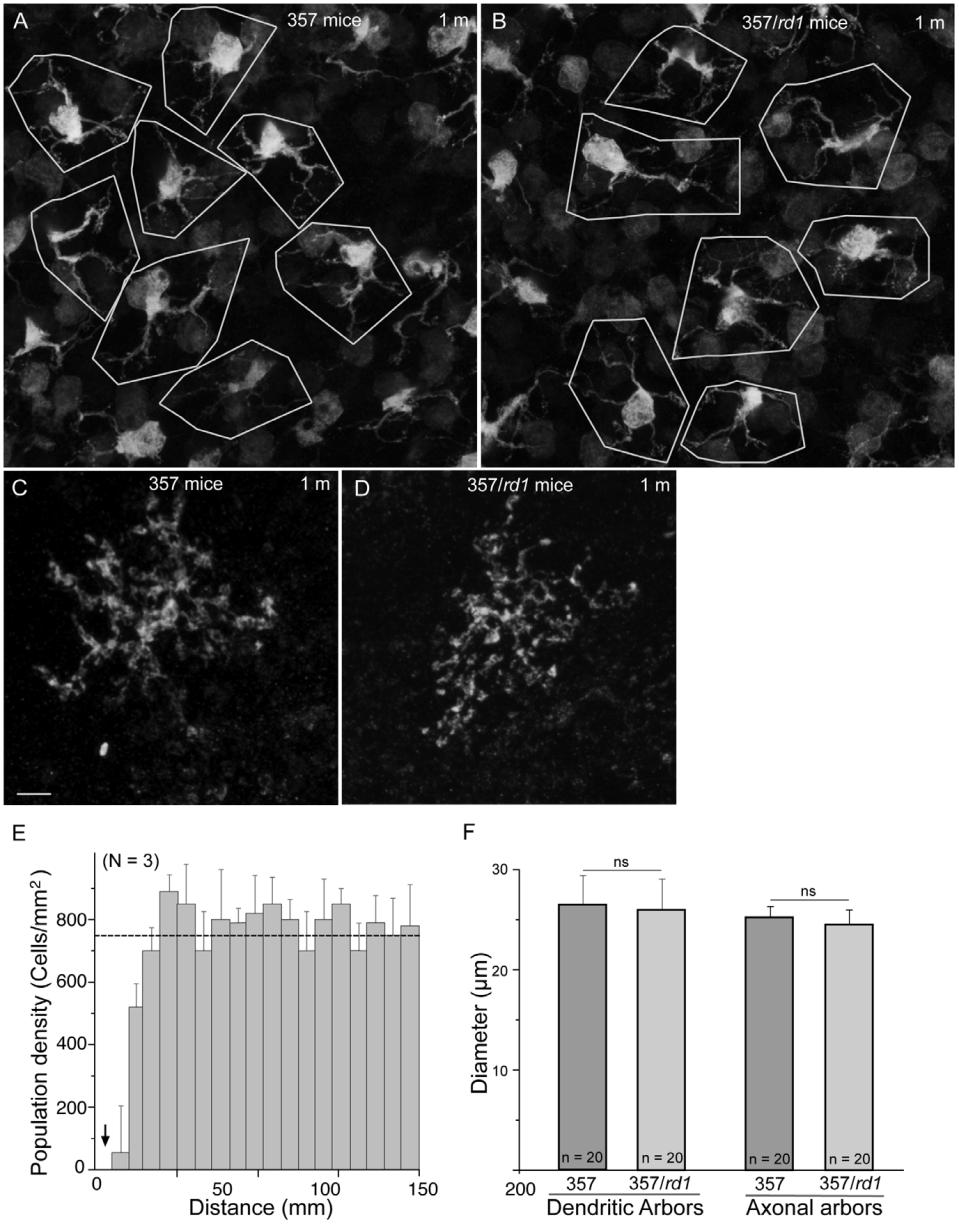
Development of dendrites, axon terminals and a regular organization of type 7 bipolar cells in *rd1* mice. **A**–**B**, The distributions of somata and the dendritic tilings of type 7 bipolar cells in the flat-mounted retinas of 357 (**A**) and 357/*rd1* (**B**) mice. The somata of type 7 bipolar cells showed regular distributions, and the dendrites of type 7 bipolar cells tiled the retinal surface with little overlap in both 357 (**A**) and 357/*rd1* (**B**) retinas. The boundaries of individual dendritic fields are shown by white polygons. Type 7 bipolar cells have similar dendritic and axonal arbors in WT (**A,** **C**) and *rd1* retinas (**B,** **D**). **E**, Analysis of the spatial distribution of type 7 bipolar cell somata in *rd1* retinas: density recovery profile. Three retinas from 3 mice were measured (mean ± s.d.). An exclusion zone is present (arrow), indicating that type 7 bipolar cells are prevented from occupying nearby positions on the retina – they form regular mosaics. Dotted line indicates the random distribution of the same number of cells. **F**, Histogram showing the average diameters of both dendritic and axonal arbors of type 7 bipolar cells. Twenty cells from 5 mice were measured in each age group (mean ± s.d.). ns, not significant. Scale bar, 5 µm.

Beginning between P8 and P10, ON cone bipolar cell dendrites invade cone terminals for forming ribbon synapse complex [22]. Indeed, we observed that the dendritic arbors of type 7 ON cone bipolar cells went to their corresponding synaptic region and were exclusively confined to the OPL (Figure 2, A, C and E, green). By P21, cone bipolar cell morphogenesis is largely complete [21] (Figure 2E, green). On the other hand, there was no sign of delay in maturation of type 7 bipolar cells in age-matched *rd1* retinas. Type 7 bipolar cell morphogenesis was largely unaltered (Figure 2, B, D and F; Figure 3, A and B, green). Type 7 bipolar cells have similar characteristic dendritic and axonal arbors at maturity in both WT and *rd1* retinas (Figure 3, A–D). Dendritic arbors were measured in flat mounted retinas of 1-mo-old control 357 animals and 1-mo-old 357/*rd1* animals. The average diameters were as follows: 357 mice: 25.37±3.33 µm; 357/*rd1* mice: 24.34±3.88 µm (Figure 3F). Dendritic arbors appeared little smaller in 357*/rd1* retinas than those in 357 retinas, but statistically no significant (P>0.05, one-way ANOVA). The same holds true for axonal arbors (Figure 3, C, D and F).

The diameters of axonal arbors in 357/*rd1* retinas were similar to the control: 25.86±1.88 µm (n = 20) and 26.31±1.11 µm (n = 20), respectively, which were not significant differences (P>0.05, one-way ANOVA).

We next investigated whether appropriate synaptic contacts between cones and type 7 cone bipolar cells were formed during the maturation of cone bipolar cells in the *rd1* retina. The synaptic ribbon profiles in the axonal terminals of cones were visualized by an antibody against C-terminal binding protein 2 (CtBP2), a RIBEYE homolog, which labels presynaptic ribbons in the axonal terminals of both photoreceptors and cone bipolar cells [24], [25]. Cone photoreceptors initiate ribbon synapse formation by invading the OPL at P4–P5 in the mouse retina [26], [27]. By P10, a high density of synaptic ribbons present in the axonal terminals of photoreceptors in the OPL of WT mice (Figure 2A, red, arrowheads), while few were observed in the OPL of *rd1* mice (Figure 2B, red, arrowheads). Magnified insets (Figure 2, A and B, red, arrowheads) illustrate a striking difference in ribbons densities between WT and *rd1* retinas. The density of synaptic ribbons was then rapidly reduced with age (Figure 2D, red, arrowheads), and was almost absent in the OPL of *rd1* retinas by P21 (Figure 2F, red, arrowheads), the time when cone bipolar cell morphogenesis is complete [21]. Our data suggested that cone photoreceptors underwent dramatically morphological remodeling and ribbon synapse complex in cone terminals was not appropriately formed during the differentiation of cone bipolar cells. Our observation is consistent with previous ultrastructural studies, which demonstrates that the synaptic connections between photoreceptors and bipolar cells are not properly formed in the *rd1* retina [20].

The somata of type 7 bipolar cells form regular mosaics across the retina surface in WT retinas (Figure 3A). The spatial distribution of type 7 bipolar cells was non-random in 357/*rd1*retinas, by visual observation (Figure 3B). This observation was quantitatively confirmed by the spatial autocorrelation, measured as the density recovery profile (DRP), which described the spatial density of homotypic cells as a function of distance from each other (Figure 3E). The presence of an exclusion zone (Figure 3E, arrow), indicated that type 7 bipolar cells were prevented from occupying nearby positions on the retina–they form regular mosaics that maximized the inter-cell distance. In fact, the regular mosaics appeared as early as at P21, the time when maturation of type 7 bipolar cells was complete (Data no shown). These results supported the hypothesis that mosaic regularity is regulated by intercellular interactions between homotypic cells and indifferent to other cell types [28], [29], [30].

In summary, type 7 cone bipolar cells in *rd1* retinas appeared to develop a mature characteristic morphology, and achieved a uniform coverage of the retinal surface, indicating that normal afferent innervation from cone photoreceptors did not play a critical role in controlling the features of axonal and dendritic morphology and mosaic regularity.

### Morphological Changes of Type 7 Bipolar Cells in rd1 Retinas

**Figure 4:**
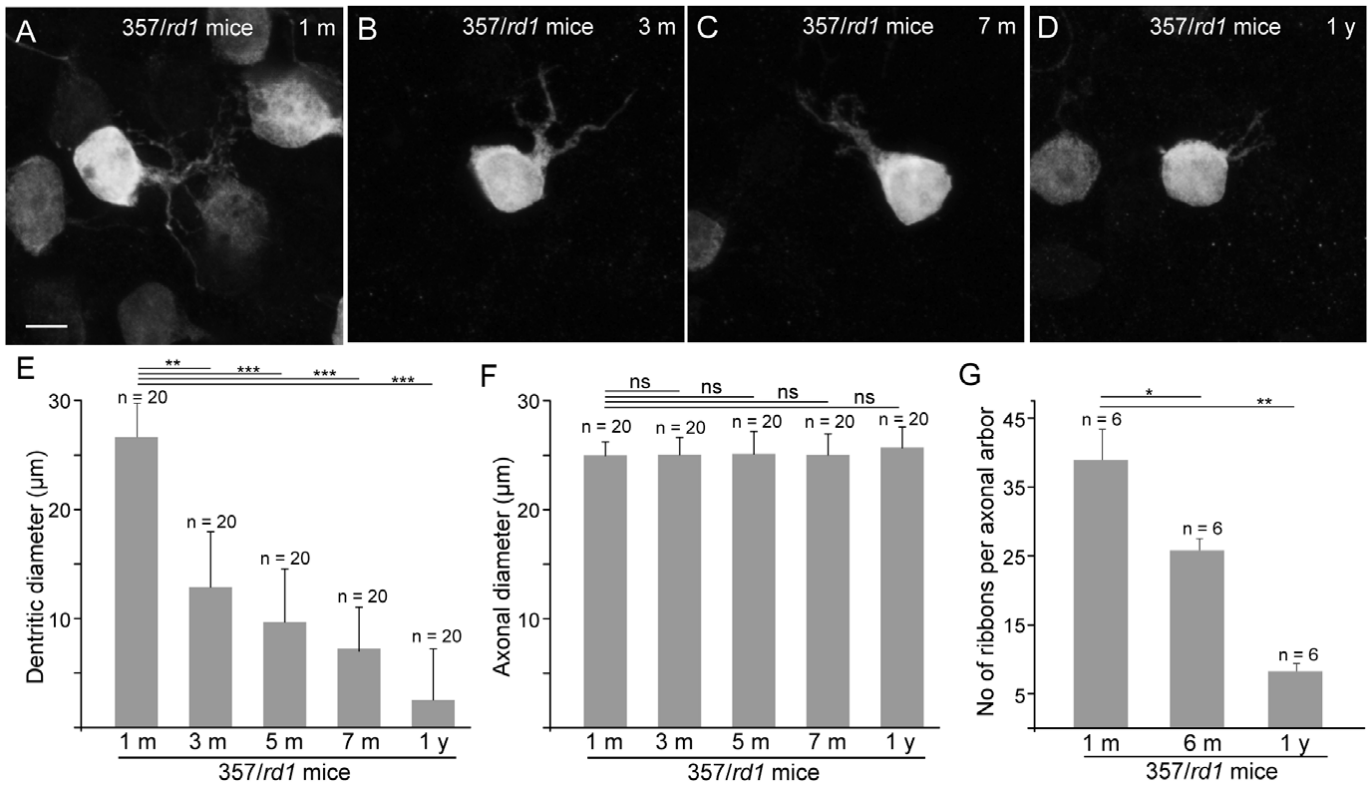
Horizontal views of the dendritic arbors of type 7 bipolar cells in flat-mounted *rd1* retinas. **A–B**, High-power images show the whole dendritic arbors of individual type 7 cone bipolar cells, illustrating a progressive dendritic retraction with growing age. **E**, Histogram showing the average diameter of dendritic arbors at different stages of degeneration. Twenty cells from 4 mice were measured in each age group (mean ± s.d.). **F**, Histogram showing the average diameter of axon arbors at different stages of degeneration. Twenty cells from 4 mice were measured in each age group (mean ± s.d.). **G**. Gradual loss of synaptic ribbons in the axonal arbors of individual type 7 cone bipolar cells in the IPL of *rd1* retinas. Six axonal terminals from 3 mice were measured in each age group (mean ± s.d.). ns, not significant, * = P<0.05, ** = P<0.01, *** = P<0.001, one-way ANOVA. Scale bar, 5 µm.

After maturation, cone bipolar cells could not maintain intact morphology for too long and became vulnerable to photoreceptor degeneration in *rd1* retinas. We found that the dendritic arbor and axonal arbor of type 7 bipolar cells responded differently to degeneration. Dendritic arbors initially underwent a rapid degeneration. The progressive dendritic retraction of type 7 bipolar cells was observed in high-power images of flat mounted retinas of different ages, where the whole dendritic processes of type 7 bipolar cells were readily revealed (Figure 4, A–D). Quantification of dendritic arbors further confirmed the progressive retractions of dendritic arbors (Figure 4E). In contrast, the axonal arbors of type 7 bipolar cells appeared to suffer no damage of any kind and remained intact for up to 1 year of age, if type 7 bipolar cells survived that long. Diameters of the axonal arbors of type 7 bipolar cells from four different ages (3 m, 5 m, 7 m and 1 yr) were measured, and the results of the quantitative analysis are summarized in histograms of Figure 4F. The average diameters of axonal arbors were 25.68±1.34 µm, 24.8±2.89 µm, 25.12±1.76 µm and 26.3±1.66 µm for 3 m, 5 m, 7 m and 1 y age, respectively, which were not significantly different from controls (P>0.05, one-way ANOVA), suggesting that the axonal arbor of type 7 bipolar cells were not sensitive to photoreceptor degeneration.

Although type 7 cone bipolar cells maintained their whole axonal arbors for up to 1 year, numbers of synaptic ribbons in the axonal arbors were gradually reduced. Axonal arbors were examined for the presence of synaptic ribbon profiles, visualized by an antibody against CtBP2. We surveyed bipolar axon arbors in cross sections at three different ages (1 m, 6 m and 1 yr). For each age, 6 terminals, which were all from dorsal retinas of 3 mice, were analyzed quantitatively (yielding in a total of 18 bipolar axon terminals) (Figure 4G). The average number of co-localized synaptic ribbons was 38.8±4.6 per axonal arbor at 1 month of age. Numbers of co-localized synaptic ribbons were reduced to 25.4±2.1 and 8±1.4 at 6 month and 12 month-old 357/*rd1* mice, respectively, a significant reduction to 34.5% and 79.4% of the number observed in 3-mo-old 357/*rd1* animals, respectively. In order to avoid some random co-localizations, we used flip analysis for some bipolar axon terminals to assess specificity of co-localization by inverting image plans along the vertical midline as previously described by us [31]. The results of these analyses were 24.5±3.2 (n = 3) at 1-month age, 16.8±2.6 (n = 3) at 6- month age and 3.9±1.8 (n = 3) at 12-month age. The number of synaptic ribbons in random co-localizations in most cases was lower than the number in actual co-localizations (Ρ<0.05, two way ANOVA), reflecting specific spatial correlation between axonal arbors and synaptic ribbons. These data suggested that there was a gradual decline in number of synaptic ribbons in axonal arbors in the first 6 months of postnatal day life, and followed by a more rapid decline over the next 6 months.

**Figure 5:**
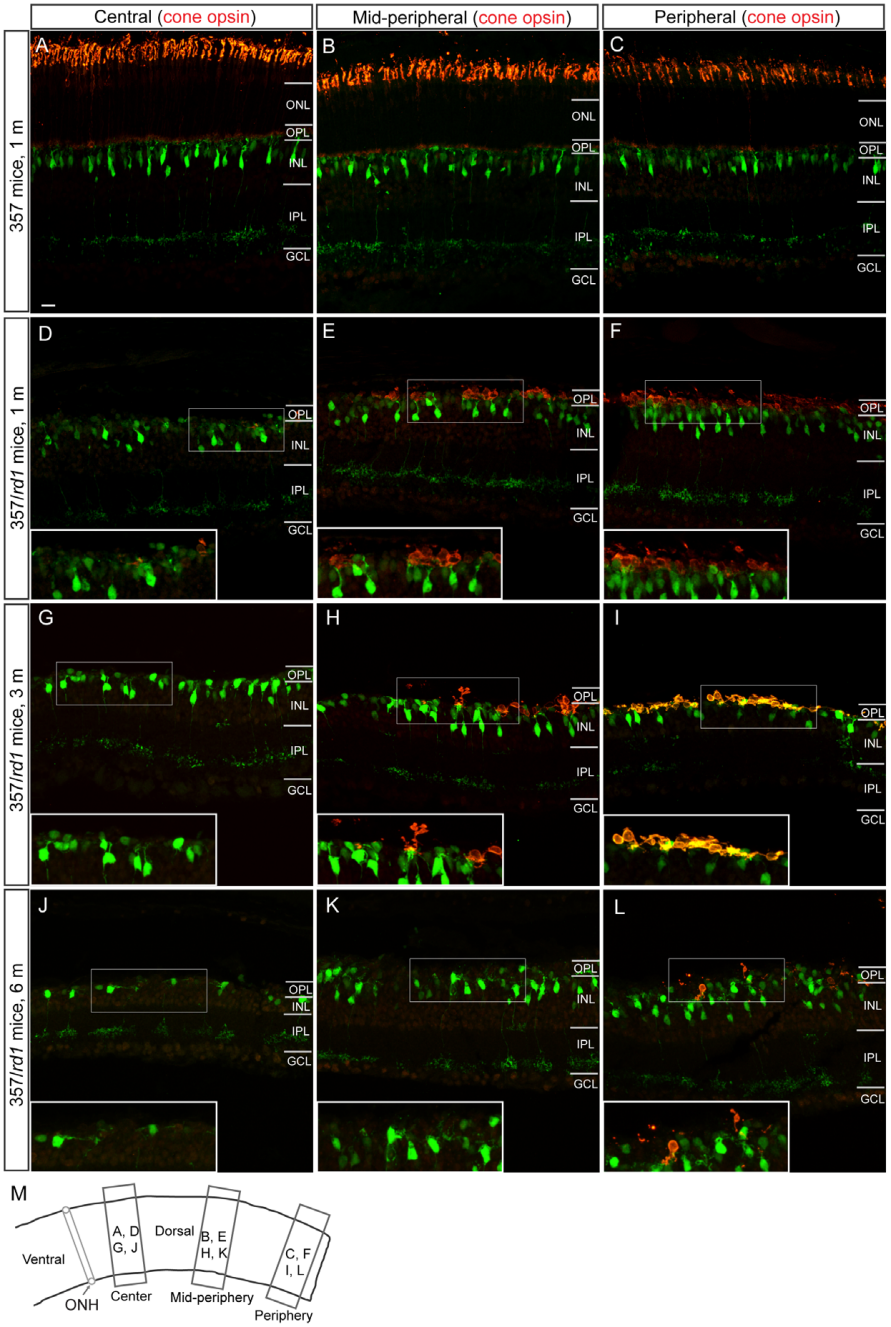
Different degeneration patterns of cone photoreceptors and type 7 bipolar cells in *rd1* mice. Counterstaining of retinal sections with an antibody against red/green opsins shows cone photoreceptors (red). **A–C**, Images were taken from three dorsal regions (**M**) of 357 mice at 1 month old, illustrating normal densities and morphologies of both type 7 cone bipolar cells (green) and cone photoreceptors (red). **D–L**, Representative images were taken from three dorsal regions of *rd1* retinas at three different ages. Insets illustrate highly magnified image from the boxed regions. Cone photoreceptors degeneration shows a central-to-peripheral progression (from **D** to **F; G** to **I; J** to **L**). Cone bipolar cell in the same regions, however, did not follow this spatiotemporal degeneration pattern (from **D** to **F; G** to **I; J** to **L**). ONL, outer nuclear layer; OPL, outer plexiform layer; INL, inner nuclear layer; IPL, inner plexiform layer; GCL, ganglion cell layer; ONH, optic nerve head. Scale bar, 20 µm.

**Figure 6:**
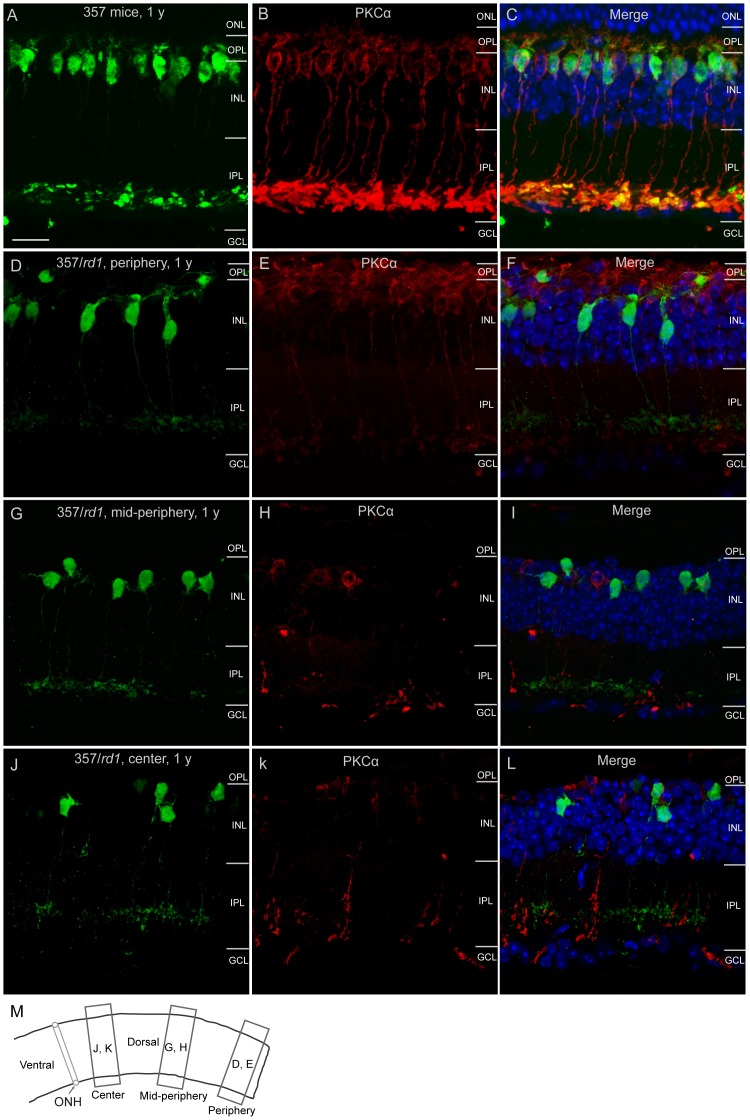
Uniform loss of type 7 cone bipolar cells across *rd1* retinas. Counterstaining of retinal sections with an antibody against PKCα shows rod bipolar cells (red). **A–C**, Images were taken from one dorsal region of a WT 357 mouse retina at 1 year old, illustrating normal densities and morphologies of type 7 cone bipolar cells (**A**) and rod bipolar cells (**B**). **C**, The merged image of **A** and **B**. Cell nuclei were stained with DAPI (blue). **D–L**, Representative images were taken from three dorsal regions of rd1 retinal sections (**M**). Rod bipolar cell degeneration showed a central-to-peripheral progression (from **K** to **H** to **E**). The degeneration of type 7 cone bipolar cell in the same retinal regions, however, did not follow this spatiotemporal pattern (from **J** to **G** to **D**), and cell loss was at the similar rate across three different eccentricities. Cell nuclei were stained with DAPI in merged images (**F**, **I**, **L**, blue). ONL, outer nuclear layer; OPL, outer plexiform layer; INL, inner nuclear layer; IPL, inner plexiform layer; GCL, ganglion cell layer; ONH, optic nerve head. Scale bar, 20 µm.

**Figure 7:**
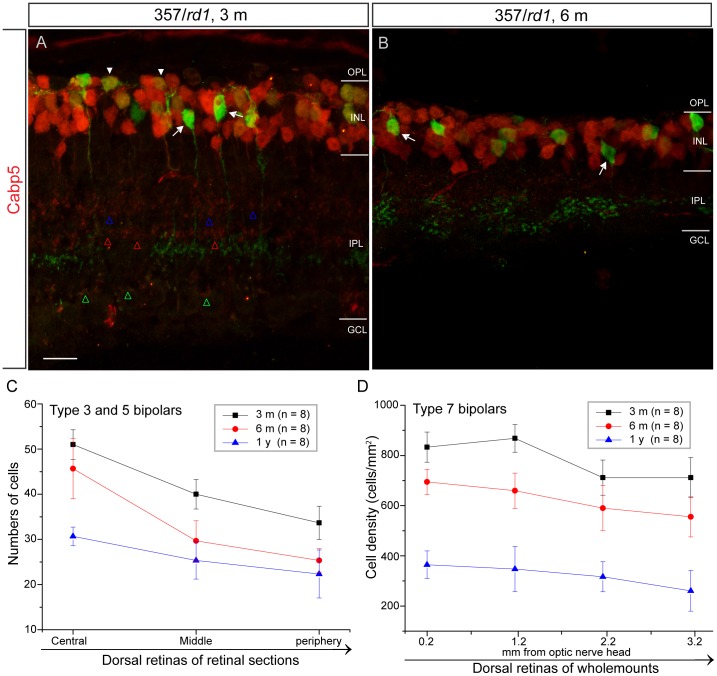
Uniform loss of type 3 OFF and 5 ON cone bipolar cells across *rd1* retinas over age. **A**–**B**, Calcium-binding protein 5 (Cabp5), is expressed by three subsets of bipolar cells (type 3 OFF and type 5 ON cone bipolar cells, and rod bipolar cells). Their axonal terminals stratified in the stratum 2 (type 3 OFF bipolars) (**A,** red, blue open arrowheads), the stratum 3 (type 5 ON bipolars) (**A,** red, red open arrowheads) and the stratum 5 (rod bipolars) (**A,** red, green open arrowheads) of the IPL, respectively. Cabp5 was not detected in type 7 cone bipolar cells (**A**, **B**, arrows), but was detected in rod bipolar cells (**A**, solid arrowheads). The density of cabp5 positive cells was decreased with age (**A**, **B**). **C**, The graph shows a gradual reduction in the densities of both type 3 OFF and type 5 ON bipolar cells over time. Cells were counted in the dorsal retinas of 8 retinal sections from 8 mice in each age group (mean ± s.d.). **D**, The graph shows a gradual decline in the densities of type 7 bipolar cells with age (mean ± s.d.). Cells were counted in the dorsal retinas of 8 flat mounted retinas along dorsoventral axe from 8 mice in each age group (mean ± s.d.). OPL, outer plexiform layer; INL, inner nuclear layer; IPL, inner plexiform layer; GCL, ganglion cell layer. Scale bar, 10 µm.

### The Unique Degeneration Pattern of Type 7 Bipolar Cells in rd1 Retinas

At global level, we studied the degeneration pattern of type 7 cone bipolar cells. Cone bipolar cells and rod bipolar cells are named for their connections with cone and rod photoreceptors, respectively. Rod bipolar cells in *rd1* retinas are dramatically affected by rapid rod photoreceptor degeneration, and follows closely the topography of rod progression [14]. Cone photoreceptors die secondarily in the *rd1* retina. The degeneration occurs in a sequential manner and always starts initially from the central retina with a central-to-peripheral progression after the major phase of rod death [9], [24], [32], [33] (Figure 5, from D to F, G to I and J to L, red). By P30, cones had disappeared from the very central retina (Figure 5D, red), and a small number of surviving cones were found to be located in extreme edge of the dorsal retina (Figure 5F, red). Numbers of surviving cones in the far peripheral dorsal retina were gradually reduced with age (Figure 5, I and L, red). Interestingly, we found that type 7 cone bipolar cells did not seem to follow the central-to-peripheral progression pattern (Figure 5, D to F, G to I and J to L, green). There was no difference in numbers of surviving type 7 bipolar cells in three regions, by visual observation. We then stained retinas with an antibody against PKCα, which specifically labels rod bipolar cells, for a comparison. Rod bipolar cells have been shown to undergo a clear central-to-peripheral progression [14]. In present study, we observed a similar generation pattern for rod bipolar cells: fewer surviving rod bipolar cells in the central retina (Figure 6K) than in the peripheral retina (Figure 6E). In the same regions, we did not observe less surviving type 7 bipolar cells in the central retina (Figure 6J) than in the peripheral retina (Figure 6D). To quantify numbers of surviving type 7 bipolar cells, we counted cells in four dorsal retinas of flat mounted retinas along dorsoventral axe directly under a microscope. Results of quantitative analysis are summarized in Figure 7D. At 3 months of age, densities of surviving type 7 bipolar cells in four regions starting from the central region were 833.3±65.2, 868±55.4, 711.8±72.5 and 711.8±80.4 cells/mm^2^, respectively (Figure 7D, black curve), which are comparable to previously published value [19]. Over the next several months, there was a gradual decline in cone bipolar cell density. Three months later at 6 months of age, reductions of 16.7%, 24%, 17.1% and 21.9% in the numbers of surviving type 7 bipolar cells in the same regions from the center to the periphery, respectively, were observed (Figure 7D, red curve). The rate of type 7 bipolar cell loss in the central, middle and peripheral retinas was not significantly different (Ρ>0.05, two-way ANOVA). Given that the early and rapid loss of cones in the central retina, one would expect to observe a greater reduction in cone bipolar cell number in this retinal region. However, we did not see a larger cell loss in the central retina than in the peripheral retina. By 1 year of age, densities of type 7 bipolar cells in our regions dropped even further to 364.6±55.5, 347.2±90.3, 316.7±60.7 and 260.4±81.3 cells/mm^2^, respectively (Figure 7D, blue curve), corresponding to reductions of 56.3%, 60%, 55.5% and 63.4% in the number observed in 3 months of age animals. There was no significant difference in the rate of type 7 bipolar cell loss in the central, middle and peripheral retinas (Ρ>0.05, two-way ANOVA). These data suggested that type 7 cone bipolar cell loss was spatially uniform across the *rd1* retina, which is different from that of cones.

To test whether the uniform cell loss pattern holds true for other subsets of cone bipolar cells in the *rd1* retina, we surveyed a panel of other types of cone bipolar cells for which specific markers are available. An antibody against calcium-binding protein 5 (Cabp5) labels type 3 OFF cone bipolar cells (Figure 7A, blue arrowheads), type 5 ON cone bipolar cells (Figure 7A, red arrowheads), and rod bipolar cells (Figure 7A, green arrowheads) in mouse retinas [34]. Cabp5 was not detected in type 7 bipolar cells (Figure 7, A and B, arrows), which is consistent with previous findings [35]. To distinguish rod bipolar cells from types 3 and 5 cone bipolar cells, we double stained retinal sections with antibodies against both Cabp5 and PKCα. Both Cabp5 positive cells and cells that were positive for both Cabp5 and PKCα in three same dorsal regions (Figure 6M) were counted. At 3 months of age, the numbers of surviving types 3 and 5 bipolar cells in three regions from the center to the periphery were 51±3.3, 40±4.5 and 33.7±3.7, respectively (Figure 7C, black line). By 6 months of age, a rapid decline in cell numbers was observed in the middle peripheral and peripheral regions (Figure 7C, red line), a reduction of 25.8% and 24.8% in the number observed at 3 months of age, respectively. A small drop in cell numbers (10.5%) was observed in the central region. By 1 year of age, however, the percentage of total cell loss was similar in three regions when compared with the numbers observed at 3 months of age: From the center to the periphery, 39.9%, 36.7% and 33.8% for the center, the mid-periphery and the periphery, respectively (Figure 7C, blue line. Ρ >0.05, two-way ANOVA), indicating that cell loss of types 3 and 5 bipolars was also uniform in *rd1* retinas. These observations further substantiated our hypothesis that the cell loss pattern of cone bipolar cells did not follow the spatiotemporal degeneration pattern of cones, and uniformity of cell loss might be a general phenomenon for all cone bipolar cell types.

## Discussion

In the present study, we anatomically characterized the morphological maturation, regular mosaic formation and degeneration pattern of type 7 cone bipolar cells in *rd1* mice. We found that type 7 cone bipolar cells achieved a uniform tiling of the retinal surface and developed mature dendritic and axonal arbors independently of afferent innervation from cone photoreceptors. However, type 7 cone bipolar cells could not maintain their normal morphology, and were succumb to degeneration in *rd1* retinas. Unexpectedly, we found the cell loss of cone bipolar cells was spatially uniform across the retina and independent of the degeneration pattern of cones. Our data suggested that both development and degeneration of cone bipolar cells were independent of cone activity.

### Disruption of Ribbon Synapse Complex in the Cone Terminals of rd1 Retinas

During differentiation, type 7 cone bipolar cells fail to form appropriate synaptic contacts with cone photoreceptors in the *rd1* retina. The reason is that bipolar cells are the last neurons to differentiate in the retina [36]. ON bipolar cells begin extending dendritic branches to the OPL and forming the classic ‘triad’ with two processes from horizontal cells at P8 [22], [26], [37]. At this time, however, cone photoreceptors already display severe morphological abnormalities in the *rd1* retina [23], [24]. It is reasonable to speculate that the synapses between cones and type 7 bipolar cells are never formed appropriately in the *rd1* retina, especially in the central retina, where the cone photoreceptors die initially. Indeed, we found that synaptic ribbons were absent in cone terminals by P10 and cone morphologies were abnormal. Our findings are consistent with previous observations that synaptogenesis is arrested in the cone terminals of *rd1* retinas, and the classic triads have never actually formed in photoreceptor terminals [20]. Similarly, ON cone bipolar cells fail to fully develop mature mGluR6 receptors in their dendritic tips in the mutant rat with retinal dystrophy [38]. Moreover, functional alterations occur at early stages of photoreceptor degeneration. Both α-waves and b-waves of the photopic electroretinogram (ERG) in *rd1* mice are profoundly affected as early as at P14, suggesting that there is an early dysfunction in the cone pathway [39], [40], [41]. Taken together, the anatomical reshaping and functional alterations from an early stage of degeneration in cone pathways indicate that appropriate synaptic contacts between cone photoreceptors and cone bipolar cells might not be formed in the *rd1* retina.

However, there are several previous reports that visually evoked responses could be observed in rd1 retinas as late as at P20–26 [42], [43], and were virtually eliminated by P28 [42]. These light-evoked responses are likely driven by spontaneous inputs from post-photoreceptor sites, such as bipolar cells and amacrine cells [44], [45], [46]. By the age of P21, the majority of cone photoreceptors had lost outer segments, which contain visual pigment molecules specialized to catch the light, as well as parts of their axon and pedicles, where photoreceptors make synaptic contacts with bipolar cells and horizontal cells (Figure 2F) [24], which make cone photoreceptors unlikely candidates for contributing to light-evoked responses. Recent evidence suggests that bipolar cells generate intrinsic rhythms that could drive downstream spontaneous activity during retinal degeneration [44], and amacrine cells are other independent oscillators [43], [47], [48], which could independently drive spike activity in RGCs. The other possibility is that intrinsically photosensitive retinal ganglion cells (ipRGCs) contribute to visual responses in *rd1* mouse retinas. Rods and cones are not the only photoreceptors in the mammalian retina. A third non-rod, non-cone photoreceptor has recently been discovered [49], [50], [51]. Melanopsin-expressing ipRGCs form a light-sensitive system separate from rods and cones are capable of autonomous phototransduction [49], [52]. Light can activate these neurons in the absence of any visual signaling from rods and cones. So far, at least five subtypes of ipRGCs with distinct morphological and physiological characteristics have been identified in the mammalian retina [53]. Since these ipRGCs are so abundant and diverse in the mammalian retina, it would be surprised to observe some RGCs are still respond to light stimuli in photoreceptor-deficient *rd1* retinas.

### The Mosaic Regularity of Type 7 Cone Bipolar Cells Resists Cone Degeneration

The present results demonstrated that the population of type 7 cone bipolar cells in the *rd1* mouse retina established the mosaic regularity and a normal dendritic and axonal morphology in the absence of normal cone photoreceptors. The somata of type 7 bipolar cells showed a nonrandom distribution of the retinal surface, suggesting that the repulsive homotypic interactions between type 7 cone bipolar cells (but not the afferent innervations from cone photoreceptors) are the underlying mechanism for the spatial organization of cone bipolar cells. Our observations are consistent with the hypothesis suggesting that mosaic regularity is regulated by intercellular interactions between homotypic cells, indifferent to other cell types [28], [29], [30], [54], [55]. Moreover, the present results also demonstrated that the sizes and stratification levels of both dendritic and axonal arbors of type 7 cone bipolar cells were not affected in *rd1* mice, indicating that the development of the morphology of cone bipolar cells was independent of cone activity, and homotypic interactions between neighboring cells largely regulate the formation of dendritic and axonal arbors. Whether other mechanisms exist to fine-tune the dendritic and axonal arbors of type 7 bipolar cells at the early or late-stages remain to be determined. One possible mechanism is local regulatory influence from horizontal cells. Horizontal cells are the first differentiated neurons to contribute processes to the nascent outer plexiform layer [26], [56], [57]. During the differentiation of cone bipolar cells, dendrites span beyond the OPL reaching the outer limiting membrane, where the dendrites likely make contacts with horizontal cell processes [21], which probably has influence on the dendritic growth of type 7 bipolar cells. Indeed, genetic removal of horizontal cells leads to smaller dendritic arbors with fewer terminal endings of type 7 bipolar cells [58]. Moreover, spontaneous depolarizations that propagate among retinal interneurons and drive RGCs to fire correlated bursts of action potentials, which is thought to be necessary for the refinement of connections between the retina and its targets [59], [60]. It is unclear, however, whether such patterns could provide the cues necessary to influence the morphological development of cone bipolar cells and mosaic regularity.

### The Vulnerability of Cone Bipolar Cells in rd1 Mice

In the present study, we first tracked the degeneration of type 7 bipolar cells in the *rd1* retina. It appeared that type 7 cone bipolar cells were susceptible to photoreceptor degeneration and died slowly and progressively. The degeneration followed a sequence similar to that of cone degeneration but began a few weeks later, and the course of degeneration was slower. Cone bipolar cells underwent dramatically morphological remodeling by initially retracting their dendrites, followed by the axon arbors and cell bodies at a slower rate. Our observation is consistent with previous reports that major reorganization of their structure occurs in the dendrites of horizontal and bipolar cells, which are the postsynaptic components to photoreceptors [14], [15], [61]. After the initial rapid degeneration in dendritic arbors, type 7 bipolar cells slowed down the pace of degeneration and retained axonal arbors and their laminar positions for many months without signs of a progressive degeneration. The processes of these remaining axon arbors were often found to contain synaptic ribbons, as visualized by antibodies against RIBEYE. When compared with their counterparts in wild type, however, the density of synaptic ribbons in axonal arbors of type 7 bipolar cells of *rd1* mice was dramatically reduced with age. These findings suggest that the bipolar-to-ganglion cell synapses might be compromised at late stages of retinal degeneration. To restore better vision, bipolar-cell based interventions, such as transgenic expression of photosensitive molecules in bipolar cells [62], [63], have to be carried out in the early stages of retinal degeneration right before the compromises of signal transmission between bipolar and ganglion cells happen. On the other hand, the therapeutic window is much shorter for photoreceptor interventions, such as, stem or progenitor cell transplantation [64] and implantation of prosthetic devices [65]. Since the success of these approaches critically depends on the normal functioning of bipolar cells at the time of transplantation, the transplantation would have to be performed before the retraction of dendritic arbors of cone bipolar cells and potential circuit corruption in the inner retina have not occurred.

Early intervention by gene therapy has demonstrated the long-term efficacy and the stability of the improvement in visual and retinal function [66], [67], suggesting the interventions have positive effect on the progression of degenerative changes.

### The Uniformity of Cone Bipolar Cell Loss Across the Retina of rd1 Mice

We did not observe the expected central-to-peripheral progression for cone bipolar cells. Instead, we found the rate and extent of cone bipolar cell loss was more or less similar at all eccentricities across the *rd1* mouse retina. This was somewhat a surprise, given the fact that early cone loss in the central retina, a greater reduction in the number of cone bipolar cell in the central retina was expected to occur, if cone bipolar cell loss followed the progression pattern of cones. Instead, we found that cone bipolar cell loss was spatially uniform across the retina. This was the conclusion when the populations of type 7 bipolar cells were surveyed. The conclusion was further substantiated by the findings that the cell loss of two other subsets of cone bipolar cells (type 3 OFF and type 5 ON) appeared to progress at a similar rate across the retina. It is thus quite possible that the uniformity of cell loss is a general phenomenon for populations of all cone bipolar cells. On the other hand, the degeneration pattern of rod bipolar cells follows closely that of rods [14]. It is not yet clear why cone bipolar cells and rod bipolar cells behave so differently in terms of their responses to photoreceptor loss. One possibility is that the dendrites of cone bipolar cells extend outside the OPL during differentiation, where these dendrites are associated with processes of horizontal cells briefly [21], which might influence the maintenance of cone bipolar cells. On the other hand, rod bipolar cells fail to develop a normal dendritic arborization [14], [20], and the association with horizontal cells may never occur during its differentiation in *rd1* mice. The other possibility is that rod bipolar cells are largely dependent on rod trophic factors for the development and survival [68]. The death of rod bipolar cell could be a result of the loss of trophic factors that is produced by healthy rods or the release of a toxin produced by dying rods, which may explain the temporal and spatial correlation of cell loss between rods and rod bipolar cells.
